# Hypopituitarism After Mild Traumatic Brain Injury: A Case Report

**DOI:** 10.7759/cureus.41282

**Published:** 2023-07-02

**Authors:** Ryan J McLoughlin, Randel L Swanson

**Affiliations:** 1 Physical Medicine and Rehabilitation, University of Pennsylvania Perelman School of Medicine, Philadelphia, USA; 2 Center for Neurotrauma, Neurodegeneration and Restoration, Corporal Michael J. Crescenz Veterans Affairs Medical Center, Philadelphia, USA

**Keywords:** neuroendocrine, testosterone replacement, hypogonadotropic hypogonadism, testosterone deficiency, testosterone, hypopituitarism, traumatic brain injury, concussion, mild traumatic brain injury (mtbi)

## Abstract

Hypopituitarism is characterized by an underactive pituitary gland and may result in growth hormone deficiency, hypothyroidism, testosterone deficiency, and/or adrenal insufficiency. Traumatic brain injury (TBI) exposure is a known risk factor for hypopituitarism. However, patients with hypopituitarism secondary to TBI exposure may go undiagnosed because the signs and symptoms of hypopituitarism can be subtle. This case report describes a 40-year-old male US military veteran who endorsed fatigue, sexual dysfunction, and weight gain several years after experiencing multiple mild TBIs during his military service. He ultimately underwent a full neuroendocrine workup that revealed low testosterone in addition to previously diagnosed hypothyroidism with a resolution of symptoms after starting testosterone therapy.

## Introduction

Traumatic brain injury (TBI) is a disruption in the brain function from a bump, blow, jolt, or penetrating injury to the head [1,2]. It is a common occurrence in modern warfare, affecting over 20% of those who served in the Army, Air Force, Navy, and Marine Corps after September 11, 2001 [3]. TBI severity classification ranges from mild to severe [1,2]. Mild TBI (mTBI), often referred to as a concussion, accounts for over 80% of all TBI exposures among service members [4] and is characterized by confusion or post-traumatic amnesia lasting up to 24 hours, a Glasgow Coma Scale of 13 to 15 in the first 24 hours after injury, or loss of consciousness lasting up to 30 minutes, in the absence of structural changes on routine neuroimaging (if acquired) [2]. The symptoms from mTBI exposure typically resolve within one to three months [1-3]. However, patients with mTBI may develop chronic symptoms, especially with repeated mTBI exposures [5].

Hypopituitarism is a deficiency of one or more of the hormones produced by the pituitary gland, including the growth hormone, thyroid-stimulating hormone (TSH), follicle-stimulating hormone (FSH), luteinizing hormone (LH), and adrenocorticotropic hormone (ACTH). In the general population, the prevalence of hypopituitarism was found to be 0.0455% in one study, making it a very rare disorder [6]. It is a potential complication after TBI exposure with a prevalence of 27.5% when measured at least five months after generalized TBI exposure [7]. An analysis of 22 individuals (mean age 28 years) with moderate to severe TBI exposure found the presence of hypopituitarism up to 178 months after the injury [8]. In addition to TBI, other risk factors for hypopituitarism include pituitary tumors; meningitis; infiltrative diseases, such as hemochromatosis; and iatrogenic injury from surgery or cranial irradiation [6]. Guidelines recommend screening for TSH, free thyroxine (T4), insulin-like growth factor-1 (IGF-1), cortisol, urinary free cortisol, FSH, LH, testosterone (in men), estradiol (in women), and prolactin three and 12 months after all moderate and severe TBI exposures, because hormone deficiencies are less likely to resolve after 12 months [9]. Screening is also recommended after mTBI exposure if the patient was admitted to the hospital for greater than 24 hours or experiences ongoing symptoms concerning for hypopituitarism three months post-injury [9].

## Case presentation

A 40-year-old male US military veteran with a past medical history of obstructive sleep apnea, hypothyroidism, hyperlipidemia, post-traumatic headaches (migraine type), and gastroesophageal reflux disease presented to the Corporal Michael J. Crescenz Veterans Affairs Medical Center’s TBI/polytrauma clinic for the evaluation and treatment of chronic neurological symptoms related to multiple TBI exposures during military service, which occurred over nine years prior to the initial TBI/polytrauma evaluation. The patient was a tank operator during his military service and reported numerous blast-related subconcussive to concussive insults characterized by disorientation and headaches immediately after firing frequent tank rounds, often with additional secondary head impacts inside the tank. He subsequently developed chronic post-traumatic migraine headaches, photophobia, binocular vision deficits, tinnitus, fatigue, irritability, and cognitive deficits (memory and concentration), along with comorbid chronic low back pain while still on active duty, the culmination of which led to a medical discharge from the military.

The TBI/polytrauma evaluation in the clinic demonstrated cognitive deficits (attention, working memory, and processing speed), photophobia, binocular vision deficits (convergence insufficiency), and post-traumatic migraine-type headaches (for which he was following with neurology and receiving treatment with a combination of botulinum toxin injections for migraine prophylaxis and oral rizatriptan as abortive therapy). The patient also presented with comorbid diagnoses of obstructive sleep apnea (managed by sleep medicine with continuous positive airway pressure therapy while sleeping) and hypothyroidism (managed by primary care with daily levothyroxine), along with depression, post-traumatic stress disorder (PTSD), and insomnia (managed by psychiatry with escitalopram, gabapentin, and trazodone).

Based on the reported mTBI exposure history coupled with the neurological examination findings detailed above, further diagnostic and therapeutic interventions were prescribed. Magnetic resonance imaging (MRI) of the brain with gradient echo (GE) and susceptibility weight imaging (SWI) sequences was ordered, which demonstrated normal brain morphology, volume, and signal intensity for his age. Comprehensive neuropsychological testing demonstrated a pattern of cognitive deficits consistent with a history of mTBI exposure(s) justifying formal cognitive rehabilitation, which the patient received in the form of speech-language pathology. He was also referred to both neuro-optometry and blind rehabilitation outpatient services (BROS), which confirmed diagnoses of photophobia, convergence insufficiency, and accommodation insufficiency. He was treated with a combination of prescription prism glasses for computer work and non-prism tinted glasses for photophobia when not performing computer work.

Despite the outpatient neurorehabilitation interventions detailed above, along with attempted control of modifiable cardiovascular risk factors, the patient reported persistent fatigue, exercise intolerance, low libido, infrequent spontaneous erections, inability to build muscle, and progressive weight gain resulting in a body mass index (BMI) of 33. Initial laboratory work-up revealed a total testosterone level of 170.1 ng/dL (reference range 300-1,000 ng/dL). He subsequently underwent a full endocrine workup two months later that confirmed the diagnosis of hypogonadotropic hypogonadism (secondary testosterone deficiency) given the presence of low total testosterone and a low/normal luteinizing hormone level (Table 1). Given the normal prolactin level and absence of other risk factors mentioned above, the presence of hypogonadotropic hypogonadism was unlikely due to the medication side effects and more likely due to his history of multiple mTBI exposures. He was started on intramuscular testosterone cypionate 200 mg every 14 days with normalization of testosterone levels to 768.6 ng/dL and significant improvement in mood, energy, libido, and exercise tolerance. He has remained stable on testosterone therapy for over 18 months without negative side effects.

**Table 1:** Laboratory evaluation of a 40-year-old man with a history of mild traumatic brain injury exposure supporting the diagnosis of hypogonadotropic hypogonadism *: Abnormal (low) total testosterone value. **: Normal TSH value, but the patient has known hypothyroidism and is appropriately treated with levothyroxine at the time of analysis. IU/L: international units per liter; mIU/L: milli-international units per liter; ng/dL: nanograms per deciliter; ng/mL: nanograms per milliliter

Test	Result	Normal range of values
Total testosterone	*140.9 ng/dL	300-1000 ng/dL
Luteinizing hormone (LH)	2.1 IU/L	1.3-8.6 IU/L
Follicle-stimulating hormone (FSH)	3.5 IU/L	1.0-21.0 IU/L
Prolactin	10.4 ng/mL	2.6-13.1 ng/mL
Thyroid-stimulating hormone (TSH)	**2.988 mIU/L	0.45-5.33 mIU/L
Prostate-specific antigen (PSA)	0.935 ng/mL	0-4 ng/mL

## Discussion

While hypopituitarism is more common after severe TBI exposures [7], it may occur after mTBI exposures [10,11]. In a systematic review, the pooled prevalence of hypopituitarism after severe and mTBI exposures was 35.3% and 16.8%, respectively [7]. An evaluation of 26 male US military veterans with a history of blast-related mTBI exposures found evidence of hypopituitarism in 11 of the 26 participants (42%) at least one year after the injury [11]. The most common deficiencies were growth hormone and testosterone [11]. A surveillance of 3409 professional US-style football players also found an association between history of concussion symptoms and self-reported low testosterone levels [10]. By contrast, an analysis of 1520 US military veterans found no relationship between history of mTBI exposure and growth hormone deficiency, low TSH, and low testosterone [12]. However, the study defined low testosterone as less than 250 ng/dL, which is lower than the American Urological Association cutoff of 300 ng/dL [13]. In addition, the study did not measure thyroxine (T4) and did not employ provocation testing for growth hormone deficiency, which may limit the interpretation of results [12].

The manifestations of hypopituitarism are nonspecific, making the diagnosis difficult in patients with multiple comorbidities (Table 2) [14]. Many symptoms of hypopituitarism, such as fatigue, mood changes, and cognitive slowing, overlap with findings after mTBI exposure [1]. The patient presented above endorsed depressed mood, low energy, and weight gain, which he partly attributed to the COVID-19 pandemic. Validated screening questionnaires for low testosterone, such as the Androgen Deficiency in the Aging Male (ADAM) questionnaire with a sensitivity of 88% for testosterone deficiency, can be used in patients with a remote history of mTBI exposure and questionable symptoms [15]. Most notably, the presence of decreased sex drive and reduced erectile strength, which was present in the patient above, should encourage neuroendocrine workup that includes TSH, free T4, IGF-1, cortisol, urinary free cortisol, FSH, LH, testosterone (in men), estradiol (in women), and prolactin [9].

**Table 2:** Clinical features of hypopituitarism

Condition	Signs and symptoms
Hypothyroidism	Fatigue, cognitive slowing, cold intolerance, hair loss, dry skin, constipation, weight gain
Growth hormone deficiency	Decreased muscle mass and strength, visceral obesity, fatigue, cognitive slowing
Testosterone deficiency	Decreased sex drive, fatigue, mood impairment, hair loss, decreased muscle mass, decreased concentration

## Conclusions

Patients with a history of mTBI exposure do not require routine lab screening for hypopituitarism. However, clinicians should have a low threshold to perform a neuroendocrine workup detailed above in patients that present with chronic fatigue, cognitive impairments, sexual dysfunction, exercise intolerance, and mood changes starting three months after mTBI exposure. Patients with initial lab abnormalities should be referred to endocrinology for confirmatory testing. Once confirmed, the treatment for hypopituitarism is relatively uncomplicated and can significantly improve quality of life by safely alleviating debilitating symptoms.
